# (*E*)-3-Dimethyl­amino-1-(2,5-dimethyl­thio­phen-3-yl)prop-2-en-1-one

**DOI:** 10.1107/S1600536812021022

**Published:** 2012-05-12

**Authors:** Mostafa M. Ghorab, Mansour S. Al-Said, Hazem A. Ghabbour, Tze Shyang Chia, Hoong-Kun Fun

**Affiliations:** a Medicinal, Aromatic and Poisonous Plants Research Center (MAPPRC), College of Pharmacy, King Saud University, PO Box 2457, Riyadh 11451, Saudi Arabia; b Department of Pharmaceutical Chemistry, College of Pharmacy, King Saud University, PO Box 2457, Riyadh 11451, Saudi Arabia; c X-ray Crystallography Unit, School of Physics, Universiti Sains Malaysia, 11800 USM, Penang, Malaysia

## Abstract

In the title compound, C_11_H_15_NOS, the 3-(dimethyl­amino)­prop-2-en-1-one unit is approximately planar [maximum deviation = 0.0975 (14) Å] and its mean plane of seven non-H atoms makes a dihedral angle of 6.96 (10)° with the thio­phene ring. In the crystal, mol­ecules are linked by pairs of C—H⋯O hydrogen bonds into inversion dimers with *R*_2_^2^(14) ring motifs. The dimers are stacked along the *c* axis through C—H⋯π inter­actions. The two methyl groups, attached to the thio­phene ring and the amino N atom, are each disordered over two orientations, with site-occupancy ratios of 0.59 (4):0.41 (4) and 0.74 (4):0.26 (4), respectively.

## Related literature

For background to and the biological activity of thio­phene derivatives, see: Ghorab *et al.* (2006 ▶); Al-Said *et al.* (2011 ▶); Shaaban *et al.* (2010 ▶); Krantz *et al.* (1990 ▶); Kikugawa & Ichino (1973 ▶); Gogte *et al.* (1967 ▶); Medower *et al.* (2008 ▶); Ghorab *et al.* (1998 ▶); Hassan *et al.* (1998 ▶). For hydrogen-bond motifs, see: Bernstein *et al.* (1995 ▶).
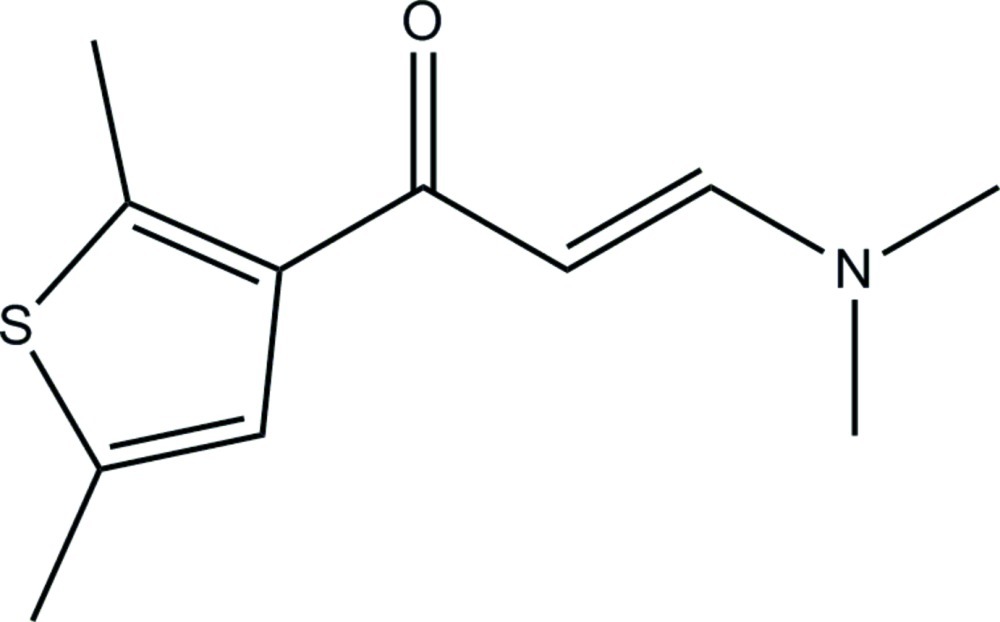


## Experimental

### 

#### Crystal data

C_11_H_15_NOS
*M* *_r_* = 209.30
Triclinic, 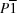

*a* = 5.9114 (2) Å
*b* = 7.5424 (2) Å
*c* = 13.9940 (4) Å
α = 81.274 (2)°
β = 88.828 (3)°
γ = 69.119 (3)°
*V* = 575.83 (3) Å^3^
*Z* = 2
Cu *K*α radiation
μ = 2.24 mm^−1^
*T* = 296 K
0.82 × 0.15 × 0.07 mm

#### Data collection

Bruker SMART APEXII CCD area-detector diffractometer
Absorption correction: multi-scan (*SADABS*; Bruker, 2009 ▶) *T*_min_ = 0.260, *T*_max_ = 0.859
7188 measured reflections
1897 independent reflections
1650 reflections with *I* > 2σ(*I*)
*R*_int_ = 0.034

#### Refinement

*R*[*F* ^2^ > 2σ(*F* ^2^)] = 0.039
*wR*(*F* ^2^) = 0.114
*S* = 1.08
1897 reflections
134 parameters
H-atom parameters constrained
Δρ_max_ = 0.16 e Å^−3^
Δρ_min_ = −0.18 e Å^−3^

### 

Data collection: *APEX2* (Bruker, 2009 ▶); cell refinement: *SAINT* (Bruker, 2009 ▶); data reduction: *SAINT*; program(s) used to solve structure: *SHELXTL* (Sheldrick, 2008 ▶); program(s) used to refine structure: *SHELXTL*; molecular graphics: *SHELXTL*; software used to prepare material for publication: *SHELXTL* and *PLATON* (Spek, 2009 ▶).

## Supplementary Material

Crystal structure: contains datablock(s) global, I. DOI: 10.1107/S1600536812021022/is5136sup1.cif

Structure factors: contains datablock(s) I. DOI: 10.1107/S1600536812021022/is5136Isup2.hkl

Supplementary material file. DOI: 10.1107/S1600536812021022/is5136Isup3.cml

Additional supplementary materials:  crystallographic information; 3D view; checkCIF report

## Figures and Tables

**Table 1:** Hydrogen-bond geometry (Å, °) *Cg*1 is the centroid of the S1/C1–C4 ring.

*D*—H⋯*A*	*D*—H	H⋯*A*	*D*⋯*A*	*D*—H⋯*A*
C10—H10*A*⋯O1^i^	0.96	2.46	3.410 (3)	172
C5—H5*B*⋯*Cg*1^ii^	0.96	2.77	3.641 (3)	152

Symmetry codes: (i) 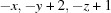; (ii) 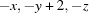.

**Fig. 1.**
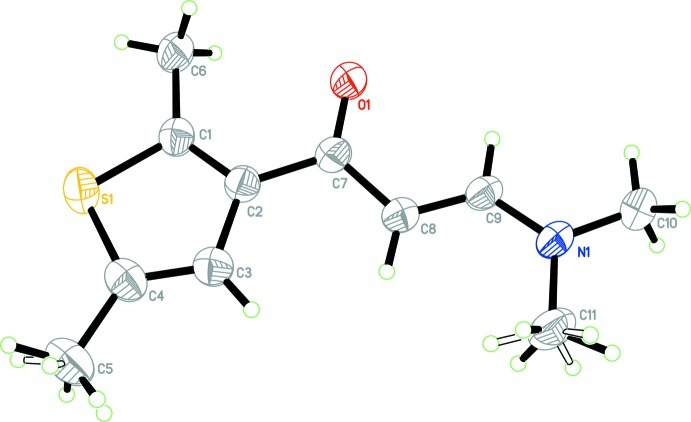
The molecular structure of the title compound with atom labels and 30% probability displacement ellipsoids.

**Fig. 2.**
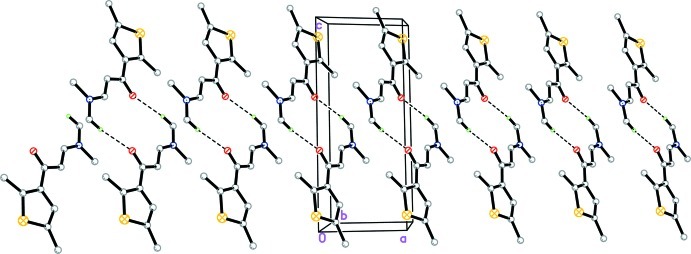
A crystal packing diagram of the title compound viewed along the b axis. The dashed lines represent the hydrogen bonds. For clarity sake, H atoms not involved in hydrogen bonding have been omitted.

## supplementary crystallographic information

### Comment

As part of a program designed to investigate the biological activity of tricyclic and tetracyclic heterocyclic systems containing a thiophene ring as the central nucleus (Ghorab *et al.*, 2006), recently we have put forward a convenient way to synthesize thiophene derivatives as anticancer agents (Al-Said *et al.*, 2011; Shaaban *et al.*, 2010). A survey of the literature showed that thiophene derivatives possess antihypertensive action (Krantz *et al.*, 1990), platelet aggregation inhibition (Kikugawa & Ichino, 1973) and antineoplastic activities (Gogte *et al.*, 1967; Medower *et al.*, 2008). In addition, several nitrogen, oxygen and sulfur-containing heterocyclic compounds incorporating thiophene residues were found to possess interesting biological properties (Ghorab *et al.*, 1998; Hassan *et al.*, 1998). In continuation of our work on the synthesis of a novel thiophene derivative which might show significant anticancer activity, the title compound was prepared and its crystal structure is now reported.

The molecular structure of the title compound is shown in Fig. 1. The mean plane of dimethylthiophene ring [S1/C1–C6; maximum deviation = 0.0180 (12) Å at atom C6] forms a dihedral angle of 6.63 (12)° with the mean plane of the rest non-H atoms [O1/N1/C7–C11; maximum deviation = 0.0975 (14) Å at atom O1]. In the molecule, the hydrogen atoms attached to atoms C5 and C11 are each disordered over two positions with site-occupancy ratios of (H5A, H5B, H5C):(H5X, H5Y, H5*Z*) = 0.59 (4):0.41 (4) and (H11A, H11B, H11C):(H11X, H11Y, H11*Z*) = 0.74 (4):0.26 (4), respectively.

In the crystal (Fig. 2), molecules are linked by pairs of intermolecular C10—H10A···O1 hydrogen bonds into inversion dimers with an *R*_2_^2^(14) ring motif (Bernstein *et al.*, 1995) and are further stacked parallel to the *a* axis. The crystal packing is further stabilized by C—H···π interaction (Table 1), involving *Cg*1 which is the centroid of S1/C1–C4 ring.

### Experimental

A mixture of 1-(2,5-dimethylthiophen-3-yl)ethanone (1.54 g, 0.01 mole) and dimethylformamide-dimethylacetal (1.19 g, 0.01 mole) in dry *N*,*N*-dimethylformamide (20 ml) was heated under reflux for 5 h. The reaction mixture was cooled and poured into ice water. The solid obtained was then recrystallized from ethanol to give the title compound. Single crystals suitable for X-ray structural analysis were obtained by slow evaporation from an *N*,*N*-dimethylformamide solution at room temperature.

### Refinement

The major parts of disordered H atoms attached to atoms C5 and C11 [(H5A, H5B, H5C) and (H11A, H11B, H11C)] were positioned geometrically, whereas the corresponding minor parts, (H5X, H5Y, H5*Z*) and (H11X, H11Y, H11*Z*) were located in a difference Fourier map. A rotating group model was used for both major and minor parts of disorders and refined using a riding model with *U*_iso_(H) = 1.5*U*_eq_(C) (C—H distance = 0.96 Å). The refined site-occupancy ratios are (H5A, H5B, H5C):(H5X, H5Y, H5*Z*) = 0.59 (4):0.41 (4) and (H11A, H11B, H11C):(H11X, H11Y, H11*Z*) = 0.74 (4):0.26 (4). The remaining H atoms were positioned geometrically (C—H = 0.93 and 0.96 Å) and refined with *U*_iso_(H) = 1.2 or 1.5*U*_eq_(C). Rotating group model was also applied to the other methyl groups in the final refinement.

### Crystal data

**Table:**

C_11_H_15_NOS	*Z* = 2
*M**_r_* = 209.30	*F*(000) = 224
Triclinic, *P*1	*D*_x_ = 1.207 Mg m^−^^3^
Hall symbol: -P 1	Cu *K*α radiation, λ = 1.54178 Å
*a* = 5.9114 (2) Å	Cell parameters from 967 reflections
*b* = 7.5424 (2) Å	θ = 3.2–67.4°
*c* = 13.9940 (4) Å	µ = 2.24 mm^−^^1^
α = 81.274 (2)°	*T* = 296 K
β = 88.828 (3)°	Plate, pink
γ = 69.119 (3)°	0.82 × 0.15 × 0.07 mm
*V* = 575.83 (3) Å^3^	

### Data collection

**Table:**

Bruker SMART APEXII CCD area-detector diffractometer	1897 independent reflections
Radiation source: fine-focus sealed tube	1650 reflections with *I* > 2σ(*I*)
Graphite monochromator	*R*_int_ = 0.034
φ and ω scans	θ_max_ = 65.0°, θ_min_ = 3.2°
Absorption correction: multi-scan (*SADABS*; Bruker, 2009)	*h* = −6→5
*T*_min_ = 0.260, *T*_max_ = 0.859	*k* = −8→8
7188 measured reflections	*l* = −16→16

### Refinement

**Table:**

Refinement on *F*^2^	Secondary atom site location: difference Fourier map
Least-squares matrix: full	Hydrogen site location: inferred from neighbouring sites
*R*[*F*^2^ > 2σ(*F*^2^)] = 0.039	H-atom parameters constrained
*wR*(*F*^2^) = 0.114	*w* = 1/[σ^2^(*F*_o_^2^) + (0.0443*P*)^2^ + 0.1016*P*] where *P* = (*F*_o_^2^ + 2*F*_c_^2^)/3
*S* = 1.08	(Δ/σ)_max_ < 0.001
1897 reflections	Δρ_max_ = 0.16 e Å^−^^3^
134 parameters	Δρ_min_ = −0.18 e Å^−^^3^
0 restraints	Extinction correction: *SHELXTL* (Sheldrick, 2008), Fc^*^=kFc[1+0.001xFc^2^λ^3^/sin(2θ)]^-1/4^
Primary atom site location: structure-invariant direct methods	Extinction coefficient: 0.010 (2)

### Special details

**Table:**

Geometry. All e.s.d.'s (except the e.s.d. in the dihedral angle between two l.s. planes) are estimated using the full covariance matrix. The cell e.s.d.'s are taken into account individually in the estimation of e.s.d.'s in distances, angles and torsion angles; correlations between e.s.d.'s in cell parameters are only used when they are defined by crystal symmetry. An approximate (isotropic) treatment of cell e.s.d.'s is used for estimating e.s.d.'s involving l.s. planes.
Refinement. Refinement of *F*^2^ against ALL reflections. The weighted *R*-factor *wR* and goodness of fit *S* are based on *F*^2^, conventional *R*-factors *R* are based on *F*, with *F* set to zero for negative *F*^2^. The threshold expression of *F*^2^ > σ(*F*^2^) is used only for calculating *R*-factors(gt) *etc*. and is not relevant to the choice of reflections for refinement. *R*-factors based on *F*^2^ are statistically about twice as large as those based on *F*, and *R*- factors based on ALL data will be even larger.

### Fractional atomic coordinates and isotropic or equivalent isotropic displacement parameters (Å^2^)

**Table:**

	*x*	*y*	*z*	*U*_iso_*/*U*_eq_	Occ. (<1)
S1	0.02985 (11)	0.64848 (8)	0.08507 (4)	0.0778 (3)	
O1	0.0467 (3)	0.8400 (2)	0.37669 (10)	0.0842 (5)	
N1	0.3675 (3)	1.2353 (2)	0.39674 (11)	0.0645 (4)	
C1	−0.0007 (3)	0.7081 (3)	0.19944 (13)	0.0608 (5)	
C2	0.1315 (3)	0.8191 (3)	0.21208 (12)	0.0572 (4)	
C3	0.2590 (4)	0.8516 (3)	0.12684 (14)	0.0665 (5)	
H3A	0.3579	0.9244	0.1232	0.080*	
C4	0.2241 (4)	0.7685 (3)	0.05249 (15)	0.0722 (5)	
C5	0.3269 (5)	0.7734 (4)	−0.04686 (17)	0.0935 (8)	
H5A	0.4761	0.7964	−0.0444	0.140*	0.59 (4)
H5B	0.2134	0.8744	−0.0910	0.140*	0.59 (4)
H5C	0.3567	0.6526	−0.0686	0.140*	0.59 (4)
H5X	0.4875	0.6789	−0.0443	0.140*	0.41 (4)
H5Y	0.3329	0.8986	−0.0688	0.140*	0.41 (4)
H5Z	0.2258	0.7459	−0.0909	0.140*	0.41 (4)
C6	−0.1564 (4)	0.6318 (3)	0.26736 (16)	0.0741 (6)	
H6A	−0.2750	0.7362	0.2930	0.111*	
H6B	−0.0568	0.5417	0.3194	0.111*	
H6C	−0.2368	0.5689	0.2330	0.111*	
C7	0.1383 (3)	0.8968 (3)	0.30347 (13)	0.0591 (5)	
C8	0.2539 (3)	1.0343 (3)	0.30342 (13)	0.0594 (5)	
H8A	0.3279	1.0684	0.2480	0.071*	
C9	0.2569 (3)	1.1156 (3)	0.38331 (13)	0.0580 (4)	
H9A	0.1705	1.0833	0.4351	0.070*	
C10	0.3421 (4)	1.3206 (3)	0.48415 (16)	0.0786 (6)	
H10A	0.2444	1.2712	0.5282	0.118*	
H10B	0.2658	1.4575	0.4684	0.118*	
H10C	0.4992	1.2896	0.5139	0.118*	
C11	0.5161 (5)	1.2903 (4)	0.3226 (2)	0.0893 (7)	
H11A	0.6385	1.1771	0.3058	0.134*	0.74 (4)
H11B	0.5917	1.3690	0.3467	0.134*	0.74 (4)
H11C	0.4161	1.3613	0.2663	0.134*	0.74 (4)
H11X	0.6789	1.2527	0.3480	0.134*	0.26 (4)
H11Y	0.4512	1.4270	0.3030	0.134*	0.26 (4)
H11Z	0.5163	1.2276	0.2678	0.134*	0.26 (4)

### Atomic displacement parameters (Å^2^)

**Table:**

	*U*^11^	*U*^22^	*U*^33^	*U*^12^	*U*^13^	*U*^23^
S1	0.0865 (4)	0.0846 (4)	0.0703 (4)	−0.0331 (3)	0.0027 (3)	−0.0296 (3)
O1	0.1196 (13)	0.1037 (12)	0.0637 (8)	−0.0794 (10)	0.0170 (8)	−0.0201 (7)
N1	0.0697 (10)	0.0645 (9)	0.0719 (10)	−0.0393 (8)	0.0060 (7)	−0.0110 (7)
C1	0.0613 (10)	0.0593 (10)	0.0623 (10)	−0.0210 (8)	−0.0024 (8)	−0.0123 (8)
C2	0.0598 (10)	0.0558 (10)	0.0570 (10)	−0.0220 (8)	−0.0008 (7)	−0.0079 (8)
C3	0.0681 (12)	0.0701 (12)	0.0638 (11)	−0.0280 (10)	0.0062 (9)	−0.0105 (9)
C4	0.0711 (12)	0.0748 (13)	0.0616 (11)	−0.0145 (10)	0.0038 (9)	−0.0119 (9)
C5	0.1003 (18)	0.1024 (19)	0.0662 (13)	−0.0218 (14)	0.0163 (12)	−0.0163 (12)
C6	0.0784 (14)	0.0821 (14)	0.0782 (13)	−0.0463 (11)	0.0042 (10)	−0.0184 (10)
C7	0.0625 (11)	0.0611 (11)	0.0599 (10)	−0.0301 (9)	0.0011 (8)	−0.0078 (8)
C8	0.0635 (11)	0.0600 (11)	0.0603 (10)	−0.0294 (8)	0.0065 (8)	−0.0084 (8)
C9	0.0583 (10)	0.0559 (10)	0.0653 (10)	−0.0289 (8)	0.0025 (8)	−0.0050 (8)
C10	0.0951 (16)	0.0787 (14)	0.0786 (13)	−0.0489 (12)	−0.0018 (11)	−0.0164 (11)
C11	0.0905 (16)	0.0933 (16)	0.1066 (17)	−0.0600 (14)	0.0237 (13)	−0.0189 (13)

### Geometric parameters (Å, º)

**Table:**

S1—C4	1.715 (2)	C5—H5Z	0.9600
S1—C1	1.7161 (19)	C6—H6A	0.9600
O1—C7	1.239 (2)	C6—H6B	0.9600
N1—C9	1.325 (2)	C6—H6C	0.9600
N1—C10	1.447 (3)	C7—C8	1.431 (3)
N1—C11	1.453 (3)	C8—C9	1.357 (3)
C1—C2	1.364 (3)	C8—H8A	0.9300
C1—C6	1.502 (3)	C9—H9A	0.9300
C2—C3	1.434 (3)	C10—H10A	0.9600
C2—C7	1.492 (3)	C10—H10B	0.9600
C3—C4	1.348 (3)	C10—H10C	0.9600
C3—H3A	0.9300	C11—H11A	0.9600
C4—C5	1.506 (3)	C11—H11B	0.9600
C5—H5A	0.9600	C11—H11C	0.9600
C5—H5B	0.9600	C11—H11X	0.9600
C5—H5C	0.9600	C11—H11Y	0.9600
C5—H5X	0.9600	C11—H11Z	0.9600
C5—H5Y	0.9600		
			
C4—S1—C1	93.45 (9)	C1—C6—H6C	109.5
C9—N1—C10	121.97 (16)	H6A—C6—H6C	109.5
C9—N1—C11	121.03 (18)	H6B—C6—H6C	109.5
C10—N1—C11	116.98 (17)	O1—C7—C8	122.18 (17)
C2—C1—C6	131.23 (18)	O1—C7—C2	119.57 (17)
C2—C1—S1	110.76 (14)	C8—C7—C2	118.26 (16)
C6—C1—S1	118.00 (14)	C9—C8—C7	120.80 (17)
C1—C2—C3	111.44 (17)	C9—C8—H8A	119.6
C1—C2—C7	123.46 (16)	C7—C8—H8A	119.6
C3—C2—C7	125.09 (17)	N1—C9—C8	128.09 (17)
C4—C3—C2	114.75 (19)	N1—C9—H9A	116.0
C4—C3—H3A	122.6	C8—C9—H9A	116.0
C2—C3—H3A	122.6	N1—C10—H10A	109.5
C3—C4—C5	129.0 (2)	N1—C10—H10B	109.5
C3—C4—S1	109.58 (15)	H10A—C10—H10B	109.5
C5—C4—S1	121.38 (19)	N1—C10—H10C	109.5
C4—C5—H5A	109.5	H10A—C10—H10C	109.5
C4—C5—H5B	109.5	H10B—C10—H10C	109.5
C4—C5—H5C	109.5	N1—C11—H11A	109.5
C4—C5—H5X	109.5	N1—C11—H11B	109.5
C4—C5—H5Y	109.5	N1—C11—H11C	109.5
H5X—C5—H5Y	109.5	N1—C11—H11X	109.5
C4—C5—H5Z	109.5	N1—C11—H11Y	109.5
H5X—C5—H5Z	109.5	H11X—C11—H11Y	109.5
H5Y—C5—H5Z	109.5	N1—C11—H11Z	109.5
C1—C6—H6A	109.5	H11X—C11—H11Z	109.5
C1—C6—H6B	109.5	H11Y—C11—H11Z	109.5
H6A—C6—H6B	109.5		
			
C4—S1—C1—C2	0.81 (15)	C1—S1—C4—C5	−179.91 (19)
C4—S1—C1—C6	−178.17 (16)	C1—C2—C7—O1	9.8 (3)
C6—C1—C2—C3	178.17 (19)	C3—C2—C7—O1	−170.80 (19)
S1—C1—C2—C3	−0.6 (2)	C1—C2—C7—C8	−170.86 (17)
C6—C1—C2—C7	−2.3 (3)	C3—C2—C7—C8	8.6 (3)
S1—C1—C2—C7	178.87 (14)	O1—C7—C8—C9	−3.3 (3)
C1—C2—C3—C4	0.1 (2)	C2—C7—C8—C9	177.32 (17)
C7—C2—C3—C4	−179.42 (18)	C10—N1—C9—C8	176.0 (2)
C2—C3—C4—C5	179.6 (2)	C11—N1—C9—C8	−2.5 (3)
C2—C3—C4—S1	0.5 (2)	C7—C8—C9—N1	175.62 (18)
C1—S1—C4—C3	−0.76 (17)		

### Hydrogen-bond geometry (Å, º)

*Cg*1 is the centroid of the S1/C1–C4 ring.

**Table:**

*D*—H···*A*	*D*—H	H···*A*	*D*···*A*	*D*—H···*A*
C10—H10*A*···O1^i^	0.96	2.46	3.410 (3)	172
C5—H5*B*···*Cg*1^ii^	0.96	2.77	3.641 (3)	152

Symmetry codes: (i) −*x*, −*y*+2, −*z*+1; (ii) −*x*, −*y*+2, −*z*.
